# Biomarkers of alcohol abuse potentially predict delirium, delirium duration and mortality in critically ill patients

**DOI:** 10.1016/j.isci.2023.108044

**Published:** 2023-09-26

**Authors:** Nikolaus Schreiber, Alexander C. Reisinger, Stefan Hatzl, Nikolaus Schneider, Laura Scholz, Markus Herrmann, Michael Kolland, Max Schuller, Alexander H. Kirsch, Kathrin Eller, Christiane Kink, Simon Fandler-Höfler, Alexander R. Rosenkranz, Gerald Hackl, Philipp Eller

**Affiliations:** 1 Department of Internal Medicine, Intensive Care Unit, Medical University of Graz, Graz, Austria; 2 Department of Internal Medicine, Division of Nephrology, Medical University of Graz, Graz, Austria; 3 Clinical Institute of Medical and Chemical Laboratory Diagnostics, Medical University of Graz, Graz, Austria; 4 Department of Neurology, Medical University Graz, Graz, Austria

**Keywords:** clinical finding, medical science

## Abstract

Carbohydrate-deficient transferrin (CDT) and the γ-glutamyltransferase-CDT derived Anttila-Index are established biomarkers for sustained heavy alcohol consumption and their potential role to predict delirium and mortality in critically ill patients is not clear. In our prospective observational study, we included 343 consecutive patients admitted to our ICU, assessed the occurrence of delirium and investigated its association with biomarkers of alcohol abuse measured on the day of ICU admission. 35% of patients developed delirium during ICU stay. We found significantly higher CDT levels (p = 0.011) and Anttila-Index (p = 0.001) in patients with delirium. CDT above 1.7% (OR 2.06), CDT per percent increase (OR 1.26, AUROC 0.75), and Anttila-Index per unit increase (OR 1.28, AUROC 0.74) were associated with delirium development in adjusted regression models. Anttila-Index and CDT also correlated with delirium duration exceeding 5 days. Additionally, Anttila-Index above 4, Anttila-Index per unit increase, and CDT per percent increase were independently associated with hospital mortality.

## Highlights

• CDT and the Anttila-Index are significantly linked to development of ICU-delirium
• CDT and the Anttila-Index are significantly associated with longer delirium duration
• CDT and the Anttila-Index also may predict hospital mortality

## Graphical abstract

**Figure:**
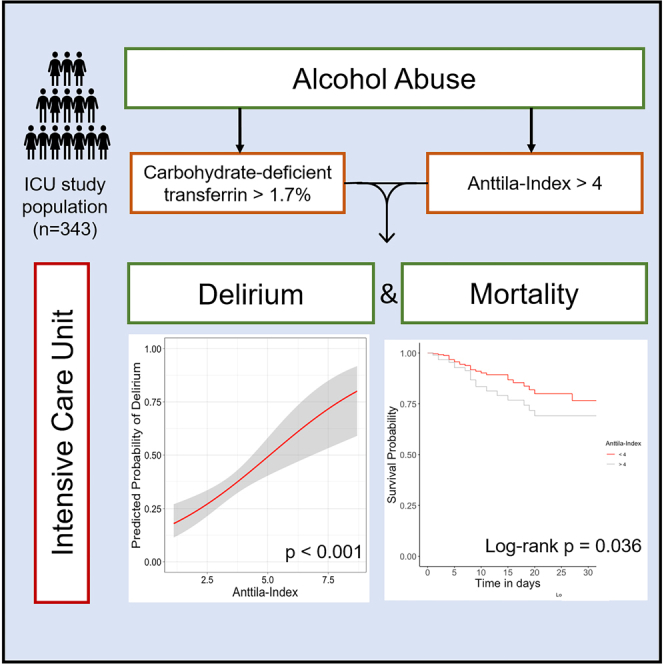

## Introduction

Delirium comprises acute brain dysfunction and represents a frequently encountered clinical entity as estimations concerning incidence of delirium in the intensive care unit (ICU) range between 20% and 80% across different institutions and study populations.^1^ Delirium is the result of a continuum of multiple predisposing and precipitating factors and poses significant risks for negative patient outcomes.^2^ To facilitate prevention of ICU-delirium, preemptive identification of patients at high risk is crucial.^3^

Alcoholism constitutes a relevant predisposing factor for development of ICU-delirium,^1^ and alcoholism related admissions to ICU are reported to be as high as 30%.^4^^,^^5^ Differentiation between alcohol-withdrawal subtype of delirium and delirium of different origin is paramount, as therapeutic consequences are directly implied.^2^^,^^6^ Benzodiazepines have been shown to be effective in the treatment of alcohol-withdrawal associated delirium, whereas their application for other indications is considered as a trigger for delirium.^7^^,^^8^ In the ICU-setting, traditional methods of diagnosing alcoholism based on self-reported surveys and questionnaires can be challenged by the acute illness, making diligent history taking often not feasible. The unreliable diagnosis of alcoholism by medical history taking can in turn obscure the opportunity to identify it as a contributing factor for ICU-delirium and may hinder the implementation of preventive measures targeting this specific risk factor.

Carbohydrate deficient transferrin (CDT) is a liver-produced variant of serum transferrin that indicates chronic heavy alcohol abuse with adequate accuracy.^9^^,^^10^ Anttila et al. proposed using CDT and γ-glutamyltransferase (γGT) measurements in a mathematical equation to enhance the diagnostic accuracy of excessive alcohol abuse disorder.^11^ No study has evaluated the relationship between these biomarkers and ICU-delirium. This study aimed to investigate the potential predictive role of these alcohol associated biomarkers in ICU-delirium.

## Results

### Baseline characteristics of study population

**Figure 1:**
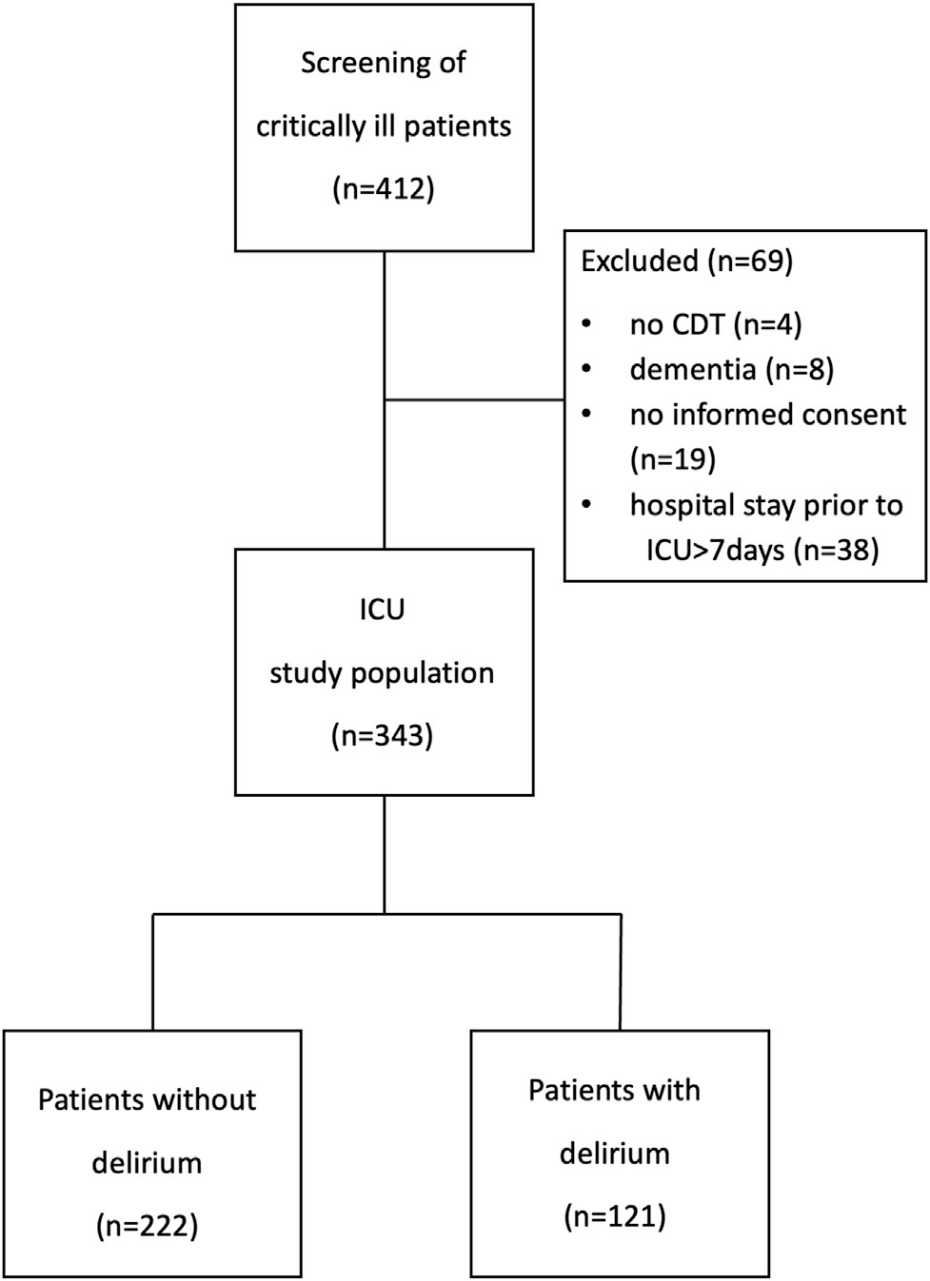
Patient flow diagram.

As shown in the patient flow diagram (Figure 1), we screened 412 critically ill patients after admission to our medical ICU and excluded 69 patients due to absence of informed consent, dementia, missing CDT values, or hospital stay longer than 7 days before ICU admission. Comatose patients, who died with a RASS score of −4 or −5 before CAM-ICU assessment was possible, were counted as “absence of informed consent.” Thus, we ultimately included 343 patients. Thereof, 35% patients (n = 121) developed delirium during their stay in the ICU. The admission diagnosis is depicted in Table 1 and demographic and clinical characteristics of our study population are detailed in Table 2.

**Table 1:** Admission diagnosis of the study population

Diagnosis on ICU admission	N = 343
AKI	13 (3.8%)
Apoplex	5 (1.5%)
Arrhythmia	5 (1.5%)
Cancer	11 (3.2%)
Cardiac arrest	9 (2.6%)
Cirrhosis	9 (2.6%)
COPD	39 (11%)
COVID 19	18 (5.2%)
Diabetic coma	14 (4.1%)
Electrolyte abnormality	18 (5.2%)
GI Bleeding	22 (6.4%)
Heart Failure	12 (3.5%)
Intoxication	47 (14%)
NSTEMI	15 (4.4%)
Other	14 (4.1%)
PAE	7 (2.0%)
Pneumonia	29 (8.5%)
Sepsis	38 (11%)
STEMI	18 (5.2%)

Proportions are depicted as percent.

AKI, Acute kidney injury; COPD, chronic obstructive pulmonary disease; COVID 19, Corona virus disease 2019; SAPS, simplified acute physiology score; TISS, therapeutic intervention scoring system; SOFA, sequential organ failure assessment; RASS, Richmond agitation-sedation scale.

**Table 2:** Demographic and clinical characteristics of the study population

Demographic and baseline characteristics	Study population [n = 343]	Patients w/o delirium [n = 222]	Patients with delirium [n = 121]	p value^a^
Age [years]	64 [52; 73]	63.5 [51; 73]	64 [52; 73]	0.988
Sex female [%]	34.7	37.4	29.8	0.193
Body mass index [kg/m^2^]	24 [22; 27]	24 [22; 27]	24 [22; 27]	0.639
SAPS3	51 [43; 62]	49 [42; 59]	57 [46; 67]	**<0.001**
TISS-28	31 [25; 34.5]	30 [24; 33]	34 [27; 38]	**<0.001**
SOFA Score	4 [2; 7]	3 [1; 5]	7 [4; 10]	**<0.001**
Age >65 years [%]	44.6	44.6	44.6	0.998
Mechanical ventilation [%]	20.4	12.2	35.6	**<0.001**
Deep sedation [RASS-5] [%]	19.8	11.3	35.6	**<0.001**
ICU-length of stay [d]	3 [2; 6]	3 [2; 4]	5 [3; 9]	**<0.001**
Hospital-length of stay [d]	9 [4; 17]	8 [4; 13]	11 [5; 22]	**<0.001**
ICU-mortality [%]	9.9	5.4	18.2	**<0.001**
Hospital-mortality [%]	14.0	9.0	23.1	**<0.001**

Median values are shown with 25^th^ and 75^th^ percentile in brackets. Statistically significant p-values are given in bold.

w/o, without; SAPS, simplified acute physiology score; TISS, therapeutic intervention scoring system; SOFA, sequential organ failure assessment; RASS, Richmond agitation-sedation scale.

a Chi-Square-Test or rank-sum test as appropriate.

Only 35% (119/343) of included patients were female with a similar sex distribution in the two subgroups with and without delirium (p = 0.193). Critically ill patients with delirium had significantly higher SAPS3, TISS28, and SOFA scores at the time of ICU admission (p < 0.001, respectively); they were more frequently in need of mechanical ventilation and deep sedation (p < 0.001 for both). Accordingly, ICU-length of stay and hospital length of stay was significantly longer in delirious patients (p < 0.001, respectively). ICU mortality was approximately three times higher in patients with ICU delirium (18% vs. 5%, p < 0.001), while the ICU-mortality rate for the general study population was roughly 10%.

The laboratory parameters at time point of ICU admission are shown in Table S1. Critically ill patients with ICU-delirium had significantly lower platelet counts (p < 0.001), lower oxygenation index (p = 0.007), and lower serum-albumin (p < 0.001). The inflammatory biomarkers C-reactive protein and procalcitonin (p = 0.005 and 0.006) as well as serum creatinine (p = 0.014) and urea (p = 0.021) were significantly elevated in delirious patients when compared to those without delirium.

### Biomarkers of alcohol abuse are associated with development of ICU-delirium

In line with our hypothesis, CDT levels were significantly higher in critically ill patients with ICU-delirium when compared to patients without delirium (p = 0.011). Indeed, 52% (33/63) of critically ill patients with CDT above 1.7% developed delirium during their stay in the ICU, whereas only 31% (88/280) of patients with a CDT level below 1.7% became delirious (p = 0.002). Furthermore, the Anttila-Index was significantly elevated in patients with delirium with a median of 3.69 in delirious patients compared to 3.22 in CAM-ICU negative patients (p = 0.001). Patients with an Anttila-Index exceeding 4 demonstrated a significantly higher incidence of delirium (49%, 45/91) in comparison to those with an Anttila-Index below 4 (30%, 76/249) (p = 0.002). (For 3 patients the Anttila-Index could not be calculated due to missing γ-glutamyltransferase values).

In our study cohort, we observed a documented history of alcohol abuse in only 16% (55/343) of individuals. However, we found that 27% of patients (91/340) had an Anttila-Index value above the cutoff, indicating the presence of excessive alcohol consumption. Cramer’s V as a measure of effect size for the correlation between documented alcohol abuse and the Anttila-Index above 4 was 0.23, indicating only moderate correlation.

This finding supports our hypothesis that alcohol abuse may be challenging to accurately diagnose in acute care settings, given the strong diagnostic performance demonstrated by the Anttila Index for detecting excessive alcohol consumption.^11^

In unadjusted binary logistic regression analysis, we found that both increase in CDT per percent point (OR of 1.34 (95% CI 1.10–1.69)), and CDT levels above the cutoff of 1.7%, (OR of 2.40 (95% CI 1.38–4.20)) were significantly associated with the development of ICU-delirium (p values of 0.008 and 0.002, respectively). Similarly, Anttila-Index per unit increase (OR of 1.47 (95% CI 1.21–1.79)), and Anttila-Index above the cutoff of 4 (OR of 2.23 (95% CI 1.36–3.65)) significantly correlated with the development of delirium in unadjusted analysis (p values of < 0.001 and 0.001, respectively). The results of unadjusted binary logistic regression analysis are depicted in Table S2.

**Figure 2:**
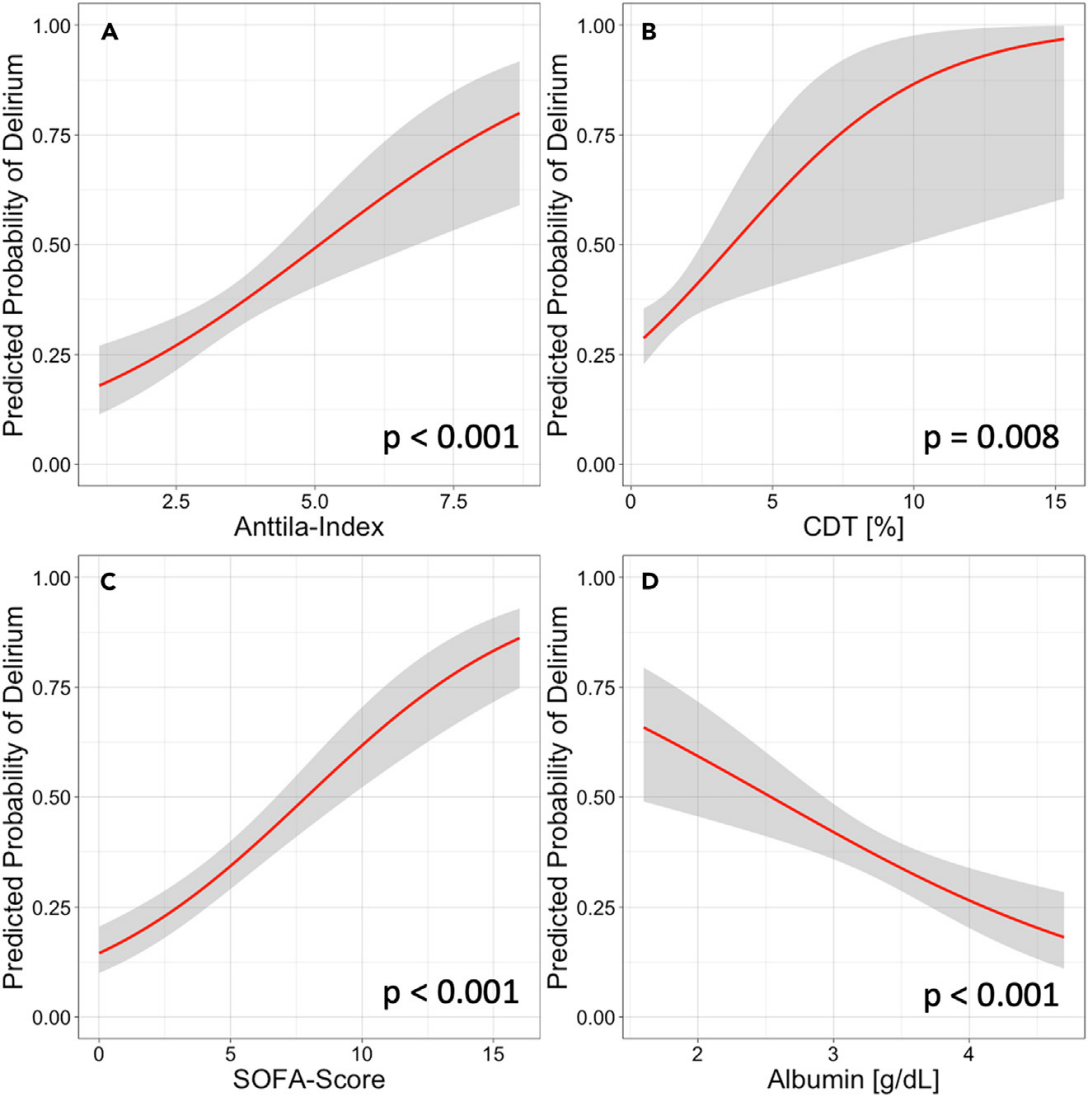
Potential predictors for development of ICU-delirium (A–D) Predicted probabilities to develop ICU delirium were derived from binary logistic regression for (A) Anttila-Index (p < 0.001), (B) carbohydrate-deficient transferrin (CDT) (p = 0.008), (C) Sequential Organ Failure Assessment (SOFA)-score (p < 0.001), and (D) serum albumin (p < 0.001), respectively. Shaded areas depict 95% confidence intervals.

**Figure 3:**
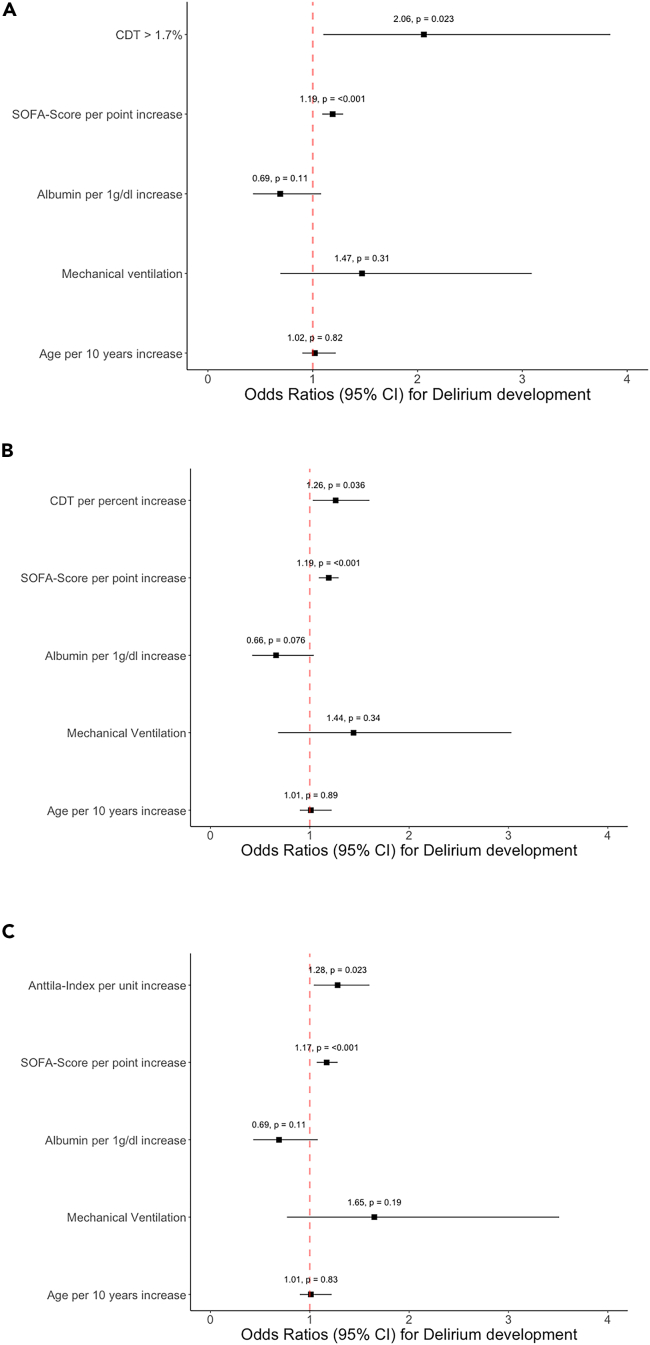
Carbohydrate-deficient transferrin and Anttila-Index are potential predictors of ICU-delirium (A–C) Multivariable logistic regression models for (A) carbohydrate-deficient transferrin (CDT) above cutoff 1.7% (OR 2.06, 95% CI 1.10–3.84, p = 0.023), (B) CDT per percent increase (OR 1.26, 95% CI 1.03–1.60, p = 0.036), and (C) Anttila-Index per unit increase (OR 1.28, 95% CI 1.04–1.60, p = 0.023) after adjustment for Sequential Organ Failure Assessment (SOFA)-score, serum albumin, mechanical ventilation and age. p values of Hosmer-Lemeshow-Tests were 0.74, 0.83, and 0.98, respectively.

Figure 2 illustrates the correlation between predicted probabilities of delirium occurrence and biomarkers of alcohol-abuse, SOFA-score, as well as serum albumin levels derived from unadjusted logistic regression analysis. Most importantly, a CDT value above 1.7%, CDT per percent increase, and Anttila-Index per unit increase remained significantly associated with delirium development in our multivariable models including the covariates SOFA-score, age, mechanical ventilation, and serum albumin (Figure 3).

### Predictive performance of CDT and Anttila-Index

The predictive performance of the CDT and Anttila-Index was evaluated using Receiver operator characteristic (ROC) analysis and the area under the receiver operator characteristic curve (AUROC). The AUROC for CDT was found to be 0.75 (95% CI: 0.69–0.81). Similarly, the AUROC for the Anttila Index was calculated to be 0.74 (95% CI: 0.68–0.80).

**Figure 4:**
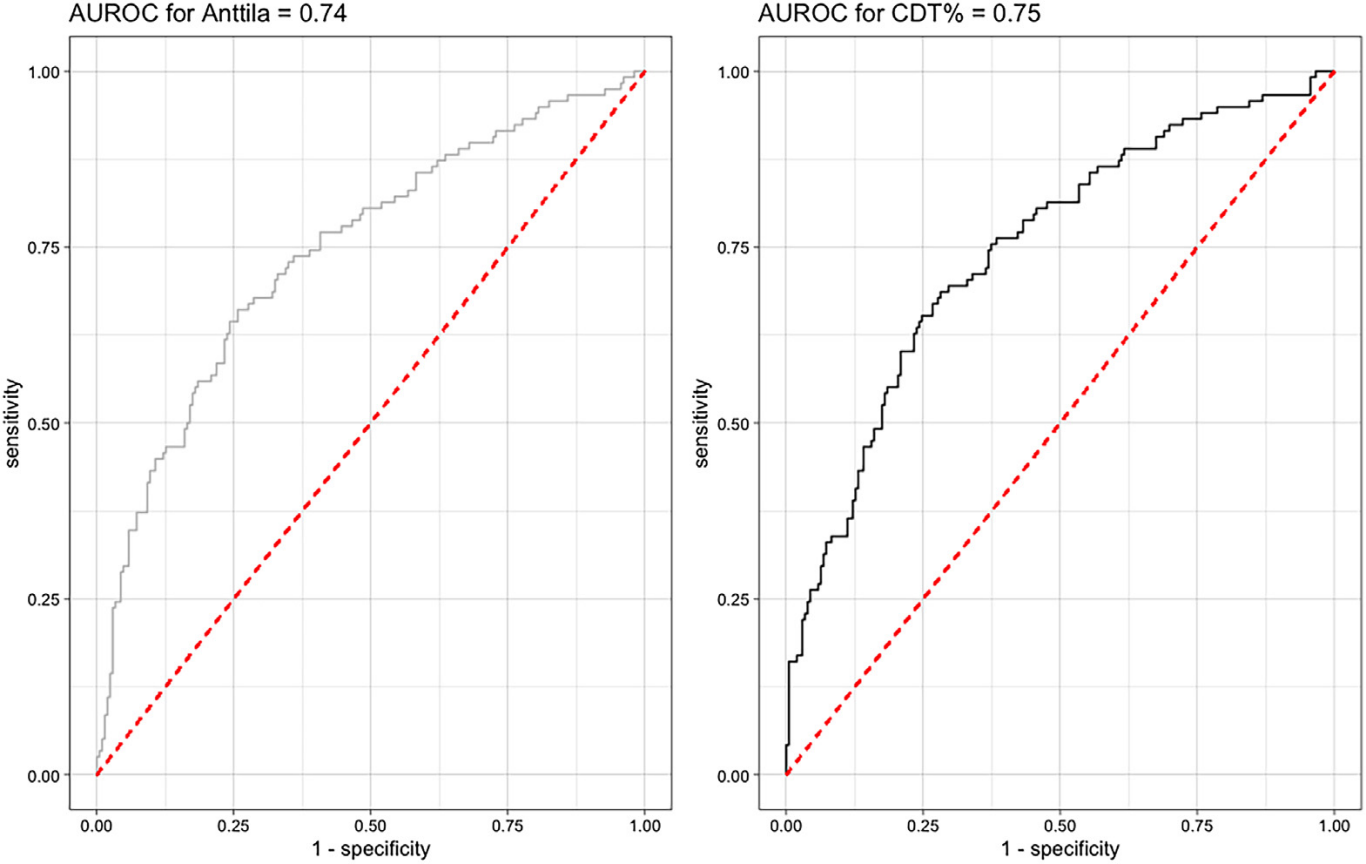
Receiver operating characteristic curves for CDT [%] and the Anttila-Index Receiver operating characteristic (ROC) curves to evaluate the predictive and discriminative ability of CDT (black line) and the Anttila-Index (gray line) for development of ICU-delirium are shown. Respective areas under the receiver operating characteristic curves (AUROC) are depicted as well. AUROC, area under the receiver operating characteristic curve; ROC, receiver operating characteristic; CDT, Carbohydrate deficient transferrin; ICU, Intensive care unit.

Optimal cutoff values were established for both the CDT and Anttila Index using the Youden Index. The CDT demonstrated an optimal threshold of 1.79%, with associated sensitivity of 0.65 and specificity of 0.75. Likewise, the Anttila Index yielded an optimal cutoff of 3.61, accompanied by sensitivity of 0.66, and specificity of 0.74 for development of ICU-delirium. The respective receiver operating characteristic (ROC) curves are outlined in Figure 4.

### Higher Anttila-Index and CDT are associated with longer delirium duration

The median duration of delirium in our cohort was 3 days (2–5 days). Based on recently published data concerning critically ill patients, which showed a range of delirium duration of 4–11 days with a median of 5 days,^12^ and the 75^th^ percentile for delirium duration of our study population at 5 days, we defined 5 days of delirium in the ICU as “prolonged ICU-delirium.” Thirty-four of 121 patients who developed delirium stayed CAM-ICU positive for 5 days or longer. Covariate adjusted logistic regression analysis in the subgroup of only CAM-ICU positive patients showed that an increase of one unit in Anttila-Index was associated with a SOFA, mechanical ventilation, age and albumin adjusted OR of 1.70 (95% CI 1.21–2.51, p = 0.004) for developing delirium lasting 5 days or more. An increase of CDT by 1% was associated with an adjusted OR of 1.34 (95% CI 1.04–1.84, p = 0.042) for developing prolonged ICU-delirium.

### Anttila-Index and CDT are linked to mortality in critically ill patients

Our study found that 14% of patients admitted to our ICU died during their stay at the hospital. Patients who developed delirium had a significantly higher mortality rate compared to patients who did not develop delirium, as demonstrated by a 23% death rate in the delirium group compared to a 9% death rate in the non-delirium group (p < 0.001). We found that Anttila-Index above 4 was associated with doubled risk of death during the hospital stay when compared to Anttila-Index below 4 in adjusted Cox proportional hazard analysis (HR 2.20, 95% CI 1.21–4.00, p = 0.010). Moreover, CDT per percent increase and Anttila-Index as continuous variables showed to be significantly associated with death during the stay at the hospital (Table 3).

**Table 3:** Adjusted hazard ratios for hospital mortality

Adjusted Cox proportional hazard model			
	Hospital mortality		
	Hazard ratio	95% Confidence interval	p value
Anttila-Index >4	2.20	1.21–4.00	**0.010**
SOFA Score per one point increase	1.11	1.03–1.20	**0.008**
Age per 10 years increase	2.16	1.63–2.84	**<0.001**
Albumin per g/dL increase	0.75	0.45–1.27	0.284
Mechanical ventilation	1.46	0.65–3.25	0.359
		**Hospital mortality**	
	**Hazard ratio**	**95% Confidence interval**	**p value**
Anttila per unit increase	1.36	1.05–1.74	**0.018**
SOFA per point increase	1.12	1.03–1.21	**0.005**
Age per 10 years increase	2.16	1.63–2.84	**<0.001**
Albumin per g/dL increase	0.74	0.44–1.25	0.265
Mechanical ventilation	1.37	0.63–2.97	0.423
		**Hospital mortality**	
	**Hazard ratio**	**95% Confidence interval**	**p value**
CDT per percent increase	1.19	1.01–1.40	**0.034**
SOFA per point increase	1.12	1.04–1.21	**0.003**
Age per 10 years increase	2.16	1.48–2.84	**<0.001**
Albumin per g/dL increase	0.72	0.42–1.21	0.210
Mechanical ventilation	1.20	0.55–2.63	0.647

Anttila-Index above 4 is independently associated with higher risk for death during stay at the hospital in cox proportional hazard analysis. (HR 2.20, 95% CI 1.21–4.00, p = 0.010). Moreover CDT (per percent increase, HR 1.19, 95% CI 1.01–1.40, p = 0.034) and Anttila-Index (per unit increase, HR 1.36, 95% CI 1.05–1.74, p = 0.018) as continuous variables were independently associated with higher risk for death. Furthermore SOFA-score and age remained significantly associated with death in all our adjusted models. Statistically significant p-values are given in bold.

CDT, carbohydrate deficient transferrin; SOFA, sequential organ failure assessment; HR, hazard ratio.

**Figure 5:**
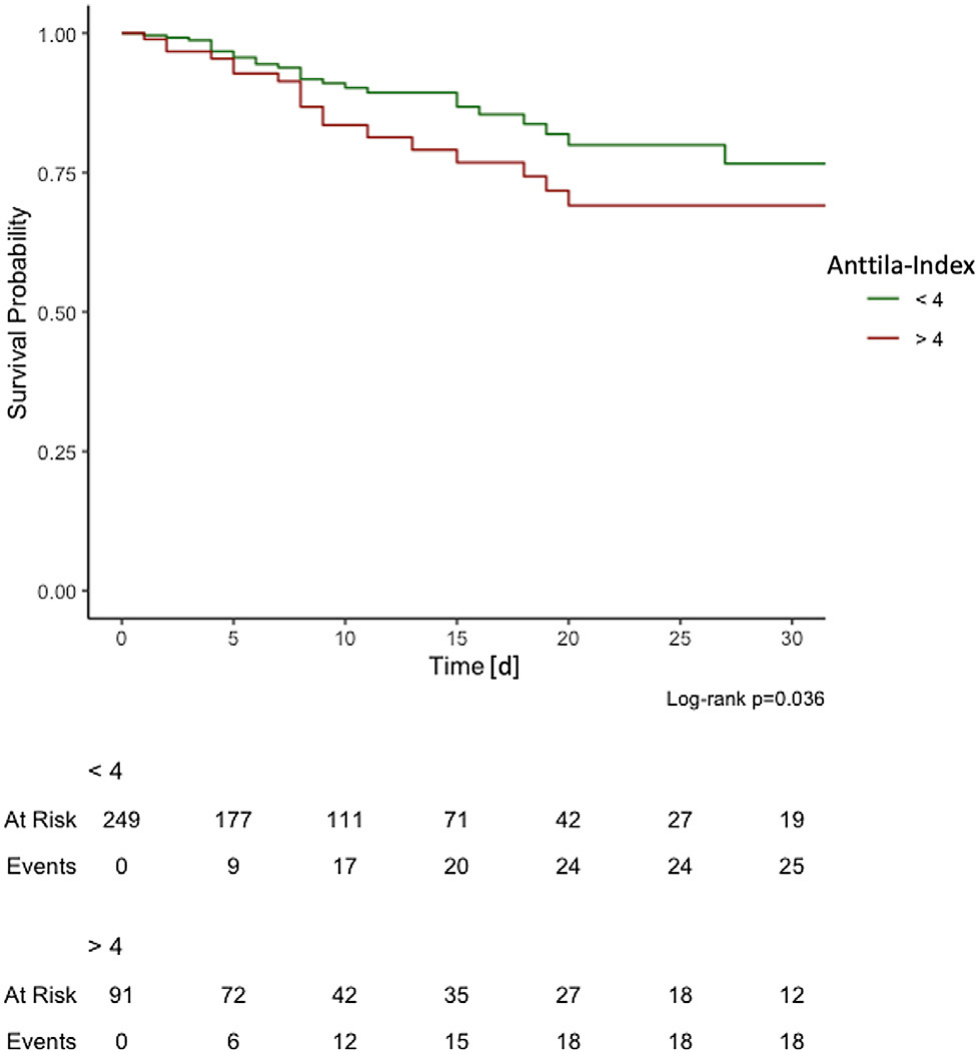
Kaplan-Meier survival curve 30-day Kaplan-Meier survival estimates for critically ill patients with Anttila-Index below 4 (black line) and Anttila-Index above 4 (gray line). Three patients were not considered because of missing values for γ-glutamyltransferase.

As depicted in the Kaplan-Meier-curve (Figure 5), the 30-day survival rate for patients with an Anttila-Index above 4 was 77%, whereas patients with an Anttila-Index below 4 had a survival rate of 69% (p = 0.036 by Log rank test).

## Discussion

The results of this prospective observational study clearly demonstrate that elevated CDT and particularly the derived Anttila-Index at time point of ICU-admission were associated with increased risk of delirium, longer delirium duration, and higher hospital mortality. We could show that these biomarkers may have a potential role in prediction of ICU-delirium and other relevant outcomes. In addition and in line with previous studies,^13^ we show that delirium causes a significant burden of disease with long ICU and hospital length of stay and most importantly high ICU and hospital mortality.

Bowman and colleagues propose specific clinical and biological subphenotyping of delirium to elucidate causal relationships between symptoms, risk factors, and biological mechanisms.^14^ Alcohol abuse is a well-known predisposing factor for the development of delirium, but there is considerable variation in the definition of alcohol abuse used in different studies.^1^^,^^14^^,^^15^^,^^16^ Additionally, assessing alcohol abuse disorder in a structured manner in critical care settings can be impractical and difficult. The complexity and multidimensional nature of alcohol abuse disorder assessments may also contribute to a lack of documentation. Our study highlights this issue, as there was a discrepancy between the proportion of patients with elevated biomarkers associated with alcohol abuse and the percentage of patients with documented histories of alcohol abuse. This discrepancy is further supported by an only moderate correlation (Cramer’s V = 0.23) between documented history of alcohol abuse and an Anttila Index above 4. This finding underscores the need for a biomarker-based approach to assess the risk of delirium in critically ill patients. While there is evidence pointing toward a higher risk for delirium development in association with use of benzodiazepines,^7^^,^^8^ a potential subphenotype of delirium identified by elevated CDT and Anttila-Index at time point of ICU-admission could potentially benefit from early use of benzodiazepines as preventive measure. However, this assumption needs to be verified in future controlled trials.

Our study harbors several strengths and limitations. RASS and CAM-ICU were performed by trained medical staff blinded to biochemical analyses of CDT to preclude influence of delirium assessment and performance of CAM-ICU. Moreover, we used prospectively collected data to assess potential association between alcohol consumption related markers and delirium occurrence. Most importantly, our findings are clinically important and relevant to intensivists, as CDT and Anttila-Index are readily available biomarkers able to identify patients at high risk for subsequent delirium development facilitating initiation of early preventive measures. Although model-based prediction of ICU-delirium has been found to have moderate to good discriminative ability,^4^^,^^17^^,^^18^^,^^19^ output from these models has been judged impractical for consequent real time action by intensivists,^2^ which further advocates a more linear approach for delirium risk assessment, as proposed in our study.

### Limitations of the study

Limitations of our study include the following aspects: (a) ICU-delirium depicts a clinical syndrome of acute brain dysfunction resulting from a complex interplay of different and partly obscure pathophysiological mechanisms, making it challenging to predict. This poses methodological problems in two dimensions. Firstly, single biomarkers have reduced discriminative performance in predicting delirium in the ICU due to the multifactorial nature of its pathogenesis and numerous confounding factors.^20^ Our findings align with these challenges, as demonstrated by the modest discriminative performance with AUROC values of 0.75 for CDT and 0.74 for the Anttila-Index. The AUROC values, however, while not achieving high levels of discrimination, still signify a capacity to differentiate patients with a heightened risk of developing ICU-delirium from those at lower risk. So, despite the intricacies inherent in the pathogenesis of delirium, our findings hold clinical relevance in identifying patients at risk. Secondly, statistical modeling to assess delirium in the ICU is challenging due to the involvement of multiple confounding factors. Therefore, we prioritized *a priori* covariate selection based on clinical judgment and evidence synthesis. To account for illness severity, which is a strong predictor of delirium development, we used the SOFA-score, a highly validated illness-severity score, as a covariate instead of relying on admission diagnosis.^14^^,^^21^^,^^22^ Additionally, we incorporated established risk factors such as mechanical ventilation, age, and serum albumin, a strong predictor of ICU-delirium that is not included in the SOFA score.^22^^,^^23^^,^^24^ We aimed to balance complexity, collinearity, and redundancy to aid interpretability of our results, hence the selection these four covariates. (b) Due to the single center, single ICU-design, our study cohort only involved critically ill medical patients which lead to lack of generalizability of our findings especially with regards to surgical patients. On the other hand, surgery and especially emergency surgery have been reported as precipitating risk factors for development of delirium per se, so risk assessment in critically ill subjects not exposed to these predisposing factors limits potential bias.^19^^,^^25^ (c) Our study was not designed to consider different delirium-psychomotor subtypes using for instance the Delirium Motor Subtype Scale (DMSS).^26^ It has, however, been proposed previously that heading away from using psychomotor subtypes for categorization of delirium might enable identification of more specific subphenotypes. Thereby, linking of precipitants with the syndrome and identification of clusters may be facilitated.^14^ This was conclusively the underlying ambition of our study.

### Conclusion

CDT and particularly the derived Anttila-Index are independently associated with development of delirium, longer delirium duration, and higher mortality in critically ill patients. Therefore, elevated CDT and Anttila-Index are not only a specific biomarker for sustained heavy alcohol consumption but may also have a role in the identification of patients at risk for delirium in the ICU setting. These findings provide a clear rationale for future interventional trials in critically ill patients to identify patients at high risk and prevent ICU-delirium.

## STAR★Methods

### Key resources table

**Table:** 

REAGENT or RESOURCE	SOURCE	IDENTIFIER
**Critical commercial assays**		

HPLC Complete Kit for CDT	ClinRep®	21000

**Software and algorithms**		

R version 4.2	R Foundation for Statistical Computing	https://www.R-project.org/
ggplot2	ggplot2: Elegant Graphics for Data Analysis. Springer-Verlag New York. ISBN 978-3-319-24277-4	https://ggplot2.tidyverse.org.
Ggsurvfit	Flexible Time-to-Event Figures	https://www.danieldsjoberg.com/ggsurvfit/
ggstatsplot	“Visualizations with statistical details: The 'ggstatsplot' approach.” Journal of Open Source Software,	doi:10.21105/joss.03167,
gtsummary	Sjoberg D, Whiting K, Curry M, Lavery J, Larmarange J (2021). “Reproducible Summary Tables with the gtsummary Package.”	doi:10.32614/RJ-2021-053
Goftes	Classical Goodness-of-Fit Tests for Univariate Distributions	https://github.com/baddstats/goftest
Tidyr	tidyr: Tidy Messy Data	https://tidyr.tidyverse.org,
Survminer	survminer: Drawing Survival Curves using 'ggplot2'	https://rpkgs.datanovia.com/survminer/index.html
pROC	Display and Analyze ROC Curves	http://expasy.org/tools/pROC/

### Resource availability

#### Lead contact

Further information and requests for resources and reagents should be directed to and will be fulfilled by the lead contact, Philipp Eller (philipp.eller@medunigraz.at)

#### Materials availability

This study did not generate new unique reagents.

### Experimental model and study participant details

#### Study population and study design

This prospective observational study was conducted in the ICU of the Department of Internal Medicine, Medical University Graz, Austria. All procedures followed were in accordance with the ethical standards of the institutional Ethics Committee of the Medical University of Graz and with the Helsinki Declaration of 1975. All patients admitted to ICU from 02/2022 until 11/2022 were screened for eligibility. Eligibility criteria were defined as follows: (1) patients admitted to medical ICU; (2) speaking German fluently; (3) age of eighteen years or older; and (4) written informed consent. Exclusion criteria were defined as follows: (1) hospital stay prior to ICU-admission longer than one week (because of time-dependent return to baseline value of CDT), (2) pre-existing mental retardation/dementia, and (3) absence of informed consent. The study protocol was approved by the Ethics Committee of the Medical University of Graz (#33–395 ex 20/21) on the 6^th^ August 2021. The study was registered in the German Clinical Trial Register DRKS as #DRKS00027015 on the 28^th^ October 2021 (https://drks.de/search/de/trial/DRKS00027015).

At the time of ICU-admission, we recorded demographic data and laboratory characteristics of these critically ill patients. All personal data were pseudonymized. The study related laboratory parameter CDT was blinded to avoid any potential influence of prior knowledge of CDT on the assessment of delirium and measurements were un-blinded after discharge of the last patient at the end of the study. All patients were of Caucasian ethnicity, and 34.7% of patients were female. Female gender had no impact on the occurrence of ICU-delirium in our study population (Table S2).

For sample size calculation we used the priorly proposed formula: n = 100 + 50i for logistic regression analysis in observational studies, where i refers to number of independent variables in the final models.^27^ We therefore aimed for 350 patients (5 independent variables in our models, see covariates in the quantification and statistical analysis section).

### Method details

Study findings were reported in accordance with the Strengthening the Reporting of Observational Studies in Epidemiology (STROBE) statement^28^ (Table S3).

#### Outcomes

Outcome variables included delirium, length of delirium, ICU-length of stay, hospital-length of stay, hospital-mortality and 30-day survival rate. Assessment of cognitive status was performed every 12 h by critical care nurses and intensivists, who were blinded to biochemical analyses of CDT. Patients were evaluated for the presence of delirium by using the Confusion Assessment Method for the ICU (CAM-ICU).^23^ Thus, delirium was defined as an acute change or fluctuation in mental status accompanied by inattention and either disorganized thinking or an altered level of consciousness (RASS scores −3 and above, and CAM-ICU positive). Comatose patients with a RASS score of −4 and −5, who did not regain consciousness during treatment in the ICU, were excluded from further analyses, as written informed consent could not be obtained from these patients, and assessment of CAM-ICU was not possible.^29^

#### Laboratory testing

All analyses were performed at the Clinical Institute for Medical and Chemical Laboratory Diagnostics at the Medical University of Graz. Full blood count analyses were performed on an XN-1000 (Sysmex Austria GmbH, Vienna, Austria), all other biochemical markers on a Cobas 8000 (Roche Diagnostics, Vienna, Austria) fully automated analyzer.

CDT was measured at time point of ICU-admission by high-performance liquid chromatography using the ClinRep HPLC Complete Kit for CDT (Recipe, Munich, Germany). This method is traceable to reference measurement procedure proposed by the International Federation of Clinical Chemistry and Laboratory Medicine Working Group on Standardization of CDT. The results are provided as the percentage of serum disialotransferrin to all transferrin fractions.^30^ In agreement with Helander et al. a cut-off value of 1.7% was used to differentiate between patients with and without excessive alcohol consumption.^30^ In addition, the *Anttila-Index* (γGT-%CDT-index) was calculated using the following equation: γGT-%CDT-index = 0.8∗ln(γGT) + 1.3∗ln(%CDT).^11^ As proposed by Anttila et al., we adopted a cutoff value of 4 for identifying excessive alcohol consumption.

#### Quantification and statistical analysis

All analyses were performed using R statistical software (v4.2.1; R Core Team 2022). Baseline demographic and clinical characteristics were presented as means and standard deviations, medians with 25th-75th percentile in brackets for continuous variables, and proportions for categorical variables. We used independent samples t-tests for normally distributed continuous variables and rank-sum tests for non-normally distributed continuous variables. Chi-Square and Fisher’s exact tests were used for categorical variables. Cramer’s V was used for assessment of effect size. We assessed the association between alcohol-associated biomarkers and delirium using unadjusted and adjusted multivariable logistic regression models. We used predicted probabilities for visualization of our models to aid in interpretation. To ensure independence and low correlation between variables, variance inflation factor analysis was conducted. The goodness of fit of our models was determined using the Hosmer-Lemeshow test. To evaluate discriminative and predictive ability of our variables we used receiver operating characteristics (ROC) analysis and calculated the area under the receiver operating curve (AUROC). To determine optimal cut-offs the Youden-Index was used.^31^

To study the relationship between biomarkers of alcohol abuse and hospital mortality, multivariable Cox proportional hazard analysis was used. Schoenfeld residuals were determined to assess the proportional hazards assumption. Kaplan-Meier analysis was conducted to compare the 30-day overall survival rate between groups with dichotomized markers of alcohol abuse and groups were compared using the log rank test. A p value of less than 0.05 was considered statistically significant.

#### Covariates

For our multivariable models, we chose *a priori* selection of potential covariates that could impact development of delirium and mortality based on prior research as well as clinical judgment. We included age, mechanical ventilation, serum albumin level and the Sequential Organ Failure (SOFA) score, with a range from 0 to 24, and higher scores indicating more severe disease and a higher risk of death. SOFA score was chosen as it adjusts for illness severity and other potential confounding covariates as platelets, oxygenation index and sedation status.^15^^,^^21^

#### Additional resources

The study was registered in the German Clinical Trial Register DRKS as #DRKS00027015 on the 28^th^ October 2021 (https://drks.de/search/de/trial/DRKS00027015).

## Data Availability

• All data reported in this paper will be shared by the lead contact upon request. • The main analysis R code is available by the lead contact upon request. • Any additional information required to reanalyze the data reported in this paper is available from the lead contact upon request.
