# Supplementary material for: Biomarkers of alcohol abuse potentially predict delirium, delirium duration and mortality in critically ill patients
Source: iScience. 2023 Sep 26;26(10):108044. doi: 10.1016/j.isci.2023.108044 (PMC10579439; doi:10.1016/j.isci.2023.108044)
Supplement: Document S1. Tables S1–S3 [file mmc1.pdf]

iScience, Volume *26*

Supplemental information

Biomarkers of alcohol abuse potentially predict delirium, delirium duration and mortality in critically ill patients

Nikolaus Schreiber, Alexander C. Reisinger, Stefan Hatzl, Nikolaus Schneider, Laura Scholz, Markus Herrmann, Michael Kolland, Max Schuller, Alexander H. Kirsch, Kathrin Eller, Christiane Kink, Simon Fandler-Höfler, Alexander R. Rosenkranz, Gerald Hackl, and Philipp Eller

Supplementary Table S1: Laboratory parameters of the study population, related to Table 2. Parameters are given as means ± SD or medians with 25th and 75th percentile in brackets. *Rank-sum test or t-test, as appropriate. Abbreviations: w/o= without, INR=international normalized ratio, CDT=carbohydrate-deficient transferrin.

Laboratory parameters	Study population [n=343]	Patients w/o delirium [n=222]	Patients with delirium [n=121]	p-value*
Leucocytes [G/L]	10.3 [7.4; 14.5]	10.5 [7.7; 14.5]	10.1 [7.0; 14.7]	0.701
Hemoglobin [g/dL]	11.9 ± 2.8	12.0 ± 2.8	11.6 ± 2.9	0.151
Platelets [G/L]	210 [154; 277]	227 [172; 296]	184 [121; 244]	**<0.001**
C-reactive protein [mg/L]	15.7 [3.7; 88.1]	11.8 [2.8; 77.6]	25.5 [6.6; 103.6]	**0.005**
Procalcitonin [ng/mL]	0.24 [0.07; 1.12]	0.19 [0.06; 0.79]	0.42 [0.11; 2.21]	**0.006**
Sodium [mmol/L]	138 [134; 140]	137 [134; 140]	138 [134; 141]	0.196
Potassium [mmol/L]	4.1 [3.8; 4.7]	4.1 [3.8; 4.6]	4.2 [3.7; 4.8]	0.767
Calcium [mmol/L]	2.22 [2.11; 2.3]	2.23 [2.15; 2.31]	2.20 [2.07; 2.29]	0.065
Phosphate [mmol/L]	1.14 [0.93; 1.40]	1.09 [0.92; 1.36]	1.19 [0.94; 1.51]	0.111
Creatinine [mg/dL]	1.01 [0.79; 1.59]	0.99 [0.79; 1.42]	1.20 [0.81; 2.04]	**0.014**
Urea [mg/dL]	41 [27; 66]	38 [27; 57]	50 [27; 84]	**0.021**
Bilirubin [mg/dL]	0.56 [0.35; 1.04]	0.55 [0.34; 0.97]	0.58 [0.36; 1.33]	0.143
γ glutamyltransferase [U/L]	48 [24; 102]	42 [24; 82]	56 [26; 156]	**0.006**
Aspartate transaminase [U/L]	35 [22; 75]	31 [20; 67]	41 [25; 92]	**0.014**
Alanine transaminase [U/L]	26 [17; 44]	26 [17; 42]	29 [16; 49]	0.688
Amylase [U/L]	22 [14; 35]	23 [15; 33]	20 [12; 41]	0.317
Creatine kinase [U/L]	110 [60; 273]	101 [58; 235.25]	130 [64.50; 367.5]	0.077
Lactate dehydrogenase [U/L]	231 [179; 346]	224.5 [177; 333]	256 [186; 365]	0.199
Prothrombin time INR	1.05 [0.97; 1.22]	1.04 [0.96; 1.18]	1.08 [1; 1.27]	**0.008**
Protein [g/dL]	6.1 ± 0.8	6.2 ± 0.8	5.9 ± 0.8	**0.010**
Albumin [g/dL]	3.4 [3.0; 3.8]	3.5 [3.1; 3.9]	3.2 [2.8; 3.7]	**<0.001**
Lactate [mmol/L]	1.1 [0.70; 1.85]	1.1 [0.70; 1.75]	1.2 [0.73; 2.15]	0.236
Oxygenation index [mm Hg]	306 [204; 400]	323 [218; 410]	283 [173; 377]	**0.007**
CDT [%]	1.19 [0.94; 1.50]	1.14 [0.93; 1.40]	1.28 [0.96; 1.79]	**0.011**
Anttila-Index	3.32 [2.67; 4.08]	3.22 [2.58; 3.84]	3.69 [2.79; 4.42]	**0.001**

Supplementary Table S2. Unadjusted logistic regression models for development of ICU-delirium, related to Figure 2 and the STAR Methods.

Binary logistic regression	ICU delirium		
	**Odds ratio**	**95% Confidence interval**	**p-value**
CDT (per one percent increase)	1.34	1.10 - 1.69	**0.008**
CDT > 1.7%	2.40	1.38 - 4.20	**0.002**
Anttila-Index	1.47	1.21 - 1.79	**<0.001**
Anttila-Index > 4	2.23	1.36 - 3.65	**0.001**
SAPS3 (per five points increase)	1.2	1.11 - 1.31	**<0.001**
TISS-28 (per one point increase)	1.07	1.04 - 1.11	**<0.001**
SOFA (per one point increase)	1.25	1.18 - 1.34	**<0.001**
Mechanical ventilation	3.98	2.32 - 6.96	**<0.001**
Deep sedation (RASS -5)	4.34	2.50 - 7.68	**<0.001**
Age (per 10 year increase)	1.02	0.89 - 1.16	0.823
Female gender	0.71	0.44 - 1.14	0.157
Procalcitonin (per 10 ng/mL increase)	1.13	0.97 - 1.36	0.141
C-reactive protein (per 50mg/L increase)	1.11	0.96 - 1.24	0.061
Creatinin (per one mg/dL increase)	1.04	0.95 - 1.15	0.389
Prothrombin time INR (per one unit increase)	1.10	0.65 - 1.82	0.700
Urea (per 10 mg/dL increase)	1.02	0.99 - 1.06	0.188
γ-glutamyltransferase (per 100 U/L increase)	1.29	1.11 - 1.56	**0.005**
Aspartate transaminase (per 50 U/L increase)	1.00	0.98 - 1.02	0.937
Albumin (per one g/dL increase)	0.50	0.34 - 0.73	**<0.001**

Supplementary Table S3. STROBE-Guideline related to the STAR Methods. Relevant text cited with line-numbers from the main manuscript in paracenteses.

	Item No	Recommendation
**Title and abstract**	1	(*a*) Indicate the study's design with a commonly used term in the title or the abstract **(Lines 1,2)**
		(*b*) Provide in the abstract an informative and balanced summary of what was done and what was found **(Lines 26-38)**
**Introduction**		
Background/rationale	2	Explain the scientific background and rationale for the investigation being reported **(Lines 40-43**
Objectives	3	State specific objectives, including any prespecified hypotheses **(Lines 62-63)**
**Methods**		
Study design	4	Present key elements of study design early in the paper **(Experimental model and study participant details section)**
Setting	5	Describe the setting, locations, and relevant dates, including periods of recruitment, exposure, follow-up, and data collection**(Experimental model and study participant details section)**
Participants	6	(*a*) Give the eligibility criteria, and the sources and methods of selection of participants. Describe methods of follow-up **(Experimental model and study participant details section)**
		(*b*) For matched studies, give matching criteria and number of exposed and unexposed **(not applicable)**
Variables	7	Clearly define all outcomes, exposures, predictors, potential confounders, and effect modifiers. Give diagnostic criteria, if applicable **(Experimental model and study participant details section)**
Data sources/ measurement	8*	For each variable of interest, give sources of data and details of methods of assessment (measurement). Describe comparability of assessment methods if there is more than one group **(Experimental model and study participant details section)**
Bias	9	Describe any efforts to address potential sources of bias **(Quantification and statistical analysis section)**
Study size	10	Explain how the study size was arrived at **(Experimental model and study participant details section)**
Quantitative variables	11	Explain how quantitative variables were handled in the analyses. If applicable, describe which groupings were chosen and why **((Quantification and statistical analysis section)**

Statistical methods	12	(*a*) Describe all statistical methods, including those used to control for confounding **(Quantification and statistical analysis section)**
		(*b*) Describe any methods used to examine subgroups and interactions **(Quantification and statistical analysis section)**
		(*c*) Explain how missing data were addressed **(Patient flow diagram(Quantification and statistical analysis section)**
		(*d*) If applicable, explain how loss to follow-up was addressed **(not applicable)**
		(*e*) Describe any sensitivity analyses **(Quantification and statistical analysis section)**
**Results**		
Participants	13*	(a) Report numbers of individuals at each stage of study—eg numbers potentially eligible, examined for eligibility, confirmed eligible, included in the study, completing follow-up, and analysed **(Patient Flow diagram, Lines 66-68)**
		(b) Give reasons for non-participation at each stage **(Patient flow diagram)**
		(c) Consider use of a flow diagram **(Patient flow diagram)**
Descriptive data	14*	(a) Give characteristics of study participants (eg demographic, clinical, social) and information on exposures and potential confounders **(Table 1, Table 2)**
		(b) Indicate number of participants with missing data for each variable of interest **(Patient Flow diagram)**
		(c) Summarise follow-up time (eg, average and total amount) **(not applicable)**
Outcome data	15*	Report numbers of outcome events or summary measures over time **(Table 1, Table2, Table 3, Figure 2, Figure3, Figure 4, Figure 5)**
Main results	16	(*a*) Give unadjusted estimates and, if applicable, confounder-adjusted estimates and their precision (eg, 95% confidence interval). Make clear which confounders were adjusted for and why they were included **(Results section), Table 2, Figure 2, Figure 3, Figure 4)**
		(*b*) Report category boundaries when continuous variables were categorized **(not applicable)**
		(*c*) If relevant, consider translating estimates of relative risk into absolute risk for a meaningful time period **(not applicable)**
Other analyses	17	Report other analyses done—eg analyses of subgroups and interactions, and sensitivity analyses **(137-138)**
**Discussion**		

Key results	18	Summarise key results with reference to study objectives **(Lines 158-164)**
Limitations	19	Discuss limitations of the study, taking into account sources of potential bias or imprecision. Discuss both direction and magnitude of any potential bias **(Limitations of the study section)**
Interpretation	20	Give a cautious overall interpretation of results considering objectives, limitations, multiplicity of analyses, results from similar studies, and other relevant evidence **(225-231)**
Generalisability	21	Discuss the generalisability (external validity) of the study results (**225-231)**
**Other information**		
Funding	22	Give the source of funding and the role of the funders for the present study and, if applicable, for the original study on which the present article is based **(242-246)**
